# Impacts of a large boreal wildfire on ground level atmospheric concentrations of PAHs, VOCs and ozone

**DOI:** 10.1016/j.atmosenv.2018.01.013

**Published:** 2018-04

**Authors:** Gregory R. Wentworth, Yayne-abeba Aklilu, Matthew S. Landis, Yu-Mei Hsu

**Affiliations:** a Environmental Monitoring and Science Division, Alberta Environment and Parks, 10th Floor 9888 Jasper Ave. NW, T5J 5C6, Edmonton, AB, Canada; b US Environmental Protection Agency, Office of Research and Development, Research Triangle Park, 27709, NC, USA; c Wood Buffalo Environmental Association, 100-330 Thickwood Blvd., T9K 1Y1, Fort McMurray, AB, Canada

**Keywords:** Wildfire, PAH, VOC, Fort McMurray, Ozone, Air quality

## Abstract

During May 2016 a very large boreal wildfire burned throughout the Athabasca Oil Sands Region (AOSR) in central Canada, and in close proximity to an extensive air quality monitoring network. This study examines speciated 24-h integrated polycyclic aromatic hydrocarbon (PAH) and volatile organic compound (VOC) measurements collected every sixth day at four and seven sites, respectively, from May to August 2016. The sum of PAHs (ΣPAH) was on average 17 times higher in fire-influenced samples (852 ng m^−3^, n = 8), relative to non-fire influenced samples (50 ng m^−3^, n = 64). Diagnostic PAH ratios in fire-influenced samples were indicative of a biomass burning source, whereas ratios in June to August samples showed additional influence from petrogenic and fossil fuel combustion. The average increase in the sum of VOCs (ΣVOC) was minor by comparison: 63 ppbv for fire-influenced samples (n = 16) versus 46 ppbv for non-fire samples (n = 90). The samples collected on August 16th and 22nd had large ΣVOC concentrations at all sites (average of 123 ppbv) that were unrelated to wildfire emissions, and composed primarily of acetaldehyde and methanol suggesting a photochemically aged air mass. Normalized excess enhancement ratios (ERs) were calculated for 20 VOCs and 23 PAHs for three fire influenced samples, and the former were generally consistent with previous observations. To our knowledge, this is the first study to report ER measurements for a number of VOCs and PAHs in fresh North American boreal wildfire plumes. During May the aged wildfire plume intercepted the cities of Edmonton (∼380 km south) or Lethbridge (∼790 km south) on four separate occasions. No enhancement in ground-level ozone (O_3_) was observed in these aged plumes despite an assumed increase in O_3_ precursors. In the AOSR, the only daily-averaged VOCs which approached or exceeded the hourly Alberta Ambient Air Quality Objectives (AAAQOs) were benzene (during the fire) and acetaldehyde (on August 16th and 22nd). Implications for local and regional air quality as well as suggestions for supplemental air monitoring during future boreal fires, are also discussed.

## Graphical abstract

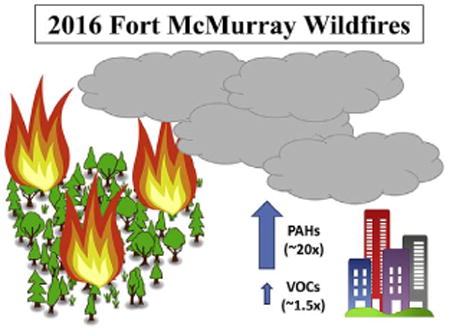

## 1. Introduction

Wildfires are a common occurrence in many regions of the world and can release significant quantities of trace gases and particulate matter to the atmosphere (Andreae and Merlet, 2001). As a result, wildfires often degrade air quality on large spatial scales leading to observable health and ecological impacts (e.g., Henderson and Johnston, 2012; Langmann et al., 2009; Stephens et al., 2014). There is strong evidence for an increasing risk of very large wildfires (> 5000 ha) as a result of climate change (Barbero et al., 2015 and references therein).

Among other pollutants, polycyclic aromatic hydrocarbons (PAHs) and volatile organic compounds (VOCs) are emitted from wildfires (e.g., Hatch et al., 2017; Jenkins et al., 1996). Some of these PAHs and VOCs are known or suspected carcinogens, and can be toxic at relatively moderate levels. Furthermore, VOCs undergo photochemical oxidation to form secondary organic aerosol (SOA) and ozone (O3), the latter of which involves a series of complex, non-linear reactions involving nitrogen oxides (NO_x_) (Seinfeld and Pandis, 2016). Numerous studies have observed significant increases in ground-level O_3_ in aged wildfire plumes due to enhanced VOCs and NO_x_ (Jaffe and Wigder, 2012; and references therein). Despite these potential effects, a review by Reisen and Brown (2006) emphasized that the majority of studies examining health impacts of wildfire smoke have focused on inhalation of fine particulate matter (PM_2.5_), and generally give less attention to potential effects of PAHs and VOCs.

The magnitude of enhancement and speciation of PAHs and VOCs near the flame front and in wildfire plumes can vary greatly since both are dependent on several factors, such as fuel loading, fuel type, burning conditions, distance to fire, and meteorology (Andreae and Merlet, 2001; Jenkins et al., 1996). For instance, Aditama (2000) measured PAH concentrations 3–65 times higher than normal during a smouldering peat wildfire haze episode in Jakarta, Indonesia. Lower molecular weight PAHs were typically enhanced more than higher molecular weight PAHs. Acetone levels were also elevated during the event, possibly due to photochemical reactions of precursor VOCs or direct wildfire emissions (Akagi et al., 2011). On the other hand, Ward et al. (2005) did not detect higher levels of PAHs in flaming mixed forest wildfire plumes in Montana. The authors attributed this to rapid photolytic degradation of PAHs during the ∼100 km transport between the fire front and sampling location. Increased concentrations of BTEX (benzene, toluene, ethylbenzene and xylene) were not observed either, possibly due to large emissions from nearby fossil fuel sources. The authors did report large increases in PM_2.5_ and phenolic compounds during the wildfire, yet modest increases in other VOCs.

A wildfire haze episode in Brunei in 1998 resulted in high concentrations of benzene (up to 25 μgm ^−3^), formaldehyde (5–22 μg m ^−3^), butyraldehyde (3–72μgm^−3^) and the sum of PAHs (ΣPAHs, 1−34 μg m^−3^, primarily as naphthalene) (Muraleedharan et al., 2000). During the event, benzene and ΣPAHs exceeded “available recommendations” for ambient concentrations, although the levels and references for these recommendations were not provided. Reinhardt and Ottmar (2004) measured several VOCs directly adjacent to a wildfire flame front to assess exposure of firefighters to air pollutants in the western United States. At the flame front, average mixing ratios of acrolein (7 ppb), benzene (16 ppb), and formaldehyde (40 ppb) were substantial.

The 2016 Fort McMurray Horse River Wildfire was one of the largest wildfires in Canada, eventually consuming ∼ 589, 600 ha (nearly 6000 km^2^) of land throughout Northern Alberta and parts of Saskatchewan. The region is dominated by northern boreal forest and wetlands with the two most abundant tree species being Jack Pine (*Pinus banksiana*) and Black Spruce (*Picea mariana*). The wildfire started on May 1, 2016 in the central part of the Athabasca Oil Sands Region (AOSR), just southwest of Fort McMurray, Alberta. By May 3 the fire began to overwhelm the city, prompting an evacuation of roughly 80,000 people from the region and causing a large number of structural fires throughout the city. Within three weeks the fire grew to more 3, 000 km^2^ (Simms, 2016) by which point the fire had moved east beyond Fort McMurray towards the Saskatchewan border. Around 2400 commercial and residential structures in Fort McMurray were burned, primarily during the period of May 4–5. Hence, it is unlikely that samples obtained outside this timeframe were significantly influenced by emissions from structural fires. Although Fort McMurray re-entry began on June 1, 2016 the wildfire was not officially under control until July 4, 2016. The AOSR encompasses ∼144, 000 km^2^ of north-eastern Alberta and contains several significant oil sands mining operations surrounded by boreal forest and wetlands. The Wood Buffalo Environmental Association (WBEA) operates a network of over 20 air quality monitoring stations throughout the AOSR, which remained operational throughout the Horse River Wildfire.

It is rare for a very large wildfire to occur adjacent to an extensive air monitoring network, where it is possible to analyse near-fire pollutant levels against well-documented historical (non-fire influenced) values. Motivated by the relatively fewer number of studies regarding the air quality implications of PAHs and VOCs from wildfires, data from the WBEA air quality monitoring network was used to examine these species during and after the Horse River Wildfire. The specific goals of this study were to:

1. Compare concentrations, speciation, and sources of PAHs and VOCs during wildfire influenced periods to non-wildfire periods (Sections 3.1 and 3.2)
2. Determine if the wildfire plume enhanced O_3_ concentrations 100s of km downwind (Section 3.3)
3. Assess the air quality implications of PAH and VOC enhancement during and after the fire by comparing observed concentrations to acute air quality metrics (Section 3.4)
4. Provide recommendations for sampling and analyses during subsequent boreal wildfires (Section 4)

## 2. Materials and methods

### 2.1. Air monitoring stations

**Fig. 1:**
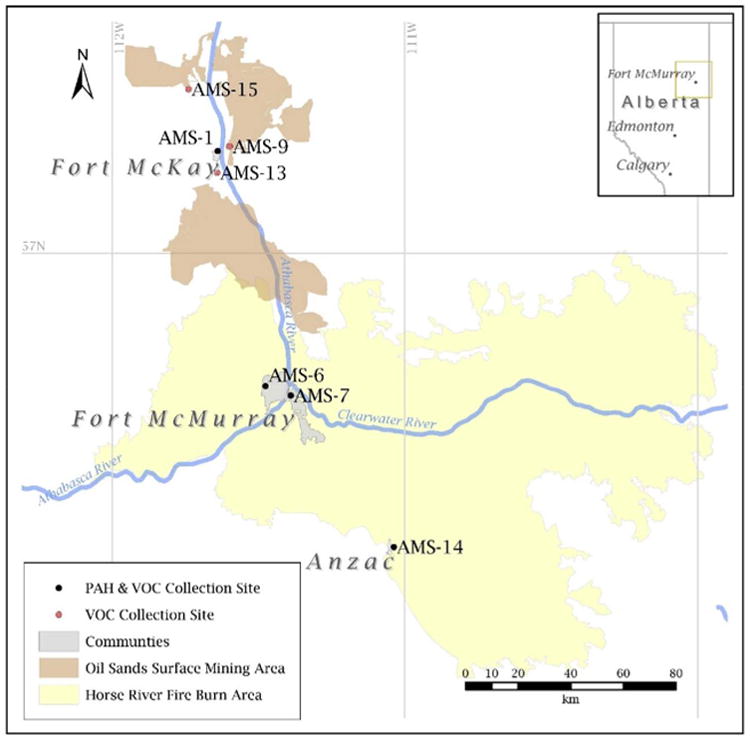
Map of the AOSR showing the 7 monitoring stations that measure PAHs and VOCs, as well as the oil sands production footprint, Fort McMurray, and Horse River Wildfire burn area.

Integrated PAH and VOC samples, as well as various continuous atmospheric measurements are routinely taken at the following air monitoring stations (AMS) throughout the AOSR: Bertha Ganter-Fort McKay (AMS 1; 111.640° W, 57.189° N), Fort McMurray Patricia McInnes (AMS 6; 111.476° W, 56.741° N), Fort McMurray Athabasca Valley (AMS 7; 111.390° W, 56.733° N), Barge Landing (AMS 9; 111.600° W, 57.198° N), Fort McKay South (AMS 13; 111.653° W, 57.149° N), Anzac (AMS 14; 111.037° W, 56.449° N), and CNRL Horizon (AMS 15; 111.740° W, 57.304° N). Fig. 1 shows the monitoring site locations, area burned by the 2016 Horse River Wildfire, and the footprint of major oil sands surface mining facilities. AMS 6 and 7 are situated in Fort McMurray (pop. ∼67, 000), and AMS 1 and 14 are in the much smaller communities of Fort McKay (pop. ∼750) and Aznac (pop. ∼550), respectively. The AMS 9, AMS 13 and AMS 15 sites are adjacent to oil sands facilities. For the duration of the intensive wildfire period (operationally defined as May 1–25, 2016) AMS 6, AMS 7 and AMS 14 were in close proximity (<10km) to the fire edge and, in some instances, surrounded by the fire.

WBEA maintains and operates the AMS sites on behalf of Alberta Environment and Parks (AEP). Quality-controlled data from the AMS sites used in this study are publicly available at: http://wbea.org/network-and-data/historical-monitoring-data (WBEA, 2016). The phase of the fire (smouldering versus flaming) is commonly investigated by calculating the modified combustion efficiency (McMeeking et al., 2009); however, this could not be calculated since none of the AMS sites monitor CO or CO_2_, except for a lone CO monitor at AMS 7.

Routine measurements of air pollutants are also conducted at air quality monitoring sites in other parts of the province on behalf of AEP. Quality-controlled data from the Edmonton-Woodcroft site (113.563° W, 53.564° N) and Lethbridge site (112.801° W, 49.716° N) are publicly available at: http://airdata.alberta.ca/aepContent/Reports/DataReports.aspx (AEP, 2016a). Edmonton (pop. ∼900,000) and Lethbridge (pop. ∼87,000) are approximately 380 km and 790 km south of Fort McMurray, respectively. These sites were chosen to examine whether plumes from the Horse River Wildfire resulted in increased ground-level O_3_ concentrations as they were advected through urban centres. Quality-controlled data for this study were used at the above sites for May 1 to August 31, 2016, inclusive.

### 2.2. PAH and VOC integrated measurements

Measurements of PAHs and VOCs at the AMS sites were conducted according to the one-in-six-day sampling schedule of the National Air Pollution Surveillance (NAPS) program. PAH samples were collected at AMS 1, AMS 6, AMS 7 and AMS 14 over a 24-h period from midnight-to-midnight every sixth day using a polyurethane foam (PUF) sampling train in a high-volume air sampler (TE-1000, Tisch Environmental) with no size cut-off. The sampling train consisted of a glass fibre filter followed by a PUF/XAD-2/PUF sandwich to capture both particulate-bound and gas-phase PAHs at a flow rate of 316 m^3^ day^−1^. Filters and sorbents were extracted with a 70:30 hexane:acetone mixture and quantified for 23 PAHs using a Gas Chromtograph/Mass Spectrometer (GC-MS, Agilent GC7890A and MS5975C) in accordance with EPA TO-13A (U.S.EPA, 1999a). Data were blank and recovery corrected. Some PAH samples could not be collected on May 6th (AMS 1, AMS 14), May 12th (AMS 14), and May 24th (AMS 1, AMS 6, AMS 14) due to limited resources and logistical difficulties during the intensive wildfire period.

A suite of 65 VOCs were quantified at all seven AMS locations in Fig. 1 using the same one-in-six-day schedule. Samples were collected over 24-h (midnight-to-midnight) using an evacuated canister and a flow of 10.0 mLmin^−1^ (TE-123, Tisch Environmental). Speciated VOCs were quantified off-line using a GC-MS. Some VOC samples were not collected during the intensive wildfire period due to restricted access: May 6th (AMS 1), May 12th (AMS 14), and May 24th (AMS 9).

Method detection limits were calculated based on the U.S. Method TO-15 (U.S.EPA, 1999b) and are presented in Supplementary Tables S1 and S2 for PAHs and VOCs, respectively. PAHs reported below the method detection limit (MDL) in the data were flagged but still included in the analyses as reported, whereas VOCs below the MDL were set to zero.

### 2.3. Continuous measurements

Ancillary continuous measurements were used to help unambiguously identify periods of wildfire influence at AMS sites in the Fort McMurray area (WBEA, 2016), as well as at the air quality monitoring sites in Edmonton and Lethbridge (AEP, 2016a). These ambient air quality data collected are subject to monitoring and reporting requirements outlined in the provincial Air Monitoring Directive (AEP, 2016b). For May to August 2016, hourly averaged quality assured data were used for PM_2.5_ mass (all sites, except AMS 9), ammonia (NH_3_; AMS 1 and AMS 6), non-methane hydrocarbons (NMHC; AMS 7 and AMS 14), total hydrocarbons (THC; AMS 9, AMS 13 and AMS 15), carbon monoxide (CO; AMS 7), NO_x_ (Woodcroft and Lethbridge), and O_3_ (Woodcroft and Lethbridge). These ancillary species at AMS locations were chosen because their ambient concentrations are greatly elevated in wildfire plumes, especially PM_2.5_ and NH_3_ (Andreae and Merlet, 2001; Benedict et al., 2017; Landis et al., 2018; Langmann et al., 2009). The NO_x_ and O_3_ data were obtained for Edmonton and Lethbridge to assess downwind O_3_ production. Information on the continuous analysers as well as their detection limits are given in Supplementary Table S3. It should be noted that the continuous NO_x_ instruments utilized in the WBEA network may suffer from a positive interference of other gaseous oxidized nitrogen species (e.g., HNO_3_, HONO, PAN); however, in these urban locations it is likely that NO_x_ dominates the gaseous NO_y_ budget.

The photometric O_3_ instruments are based on UV absorption at 254 nm, and suffered from an obvious large positive interference (e.g., nocturnal O_3_ > 250 ppbv when NO > 15ppbv) at AMS sites during periods of intense wildfire smoke which prohibited a near-flame front analysis of O_3_ concentrations (Landis et al., 2018). This phenomenon likely results from other specie(s) in the plume that absorb light at 254 nm, such as nitro- or oxidized aromatics (Dunlea et al., 2006).

### 2.4. HYSPLIT back-trajectories

Potential wildfire plumes at the Edmonton and Lethbridge sites were identified with PM_2.5_ mass measurements. The Hybrid Single-Particle Lagrangian Integrated Trajectory (HYSPLIT) model was then used to compute air parcel back-trajectories to aid in confirmation of the Horse River Wildfire influenced periods (Stein et al., 2015). Back-trajectory simulations were calculated using NARR (North American Regional Reanalysis) meteorology with 32-km resolution at air parcel arrival heights of 100 m and 500 m. For days potentially influenced by wildfires (e.g., large PM_2.5_ spikes) the model was run backwards for 120 h starting at the peak of PM_2.5_.

NASA's Fire Information Resource Managements System (FIRMS) web fire mapper tool was used to either confirm or refute the presence of wildfires along the HYSPLIT back-trajectories (NASA, 2016). The web fire mapper tool provides near-real time and historical maps of wildfires at 1 km^2^ resolution using satellite-based instruments (MODIS and VIIRS). A combination of *in situ* measurements, HYSPLIT and FIRMS determined whether a particular period at Edmonton or Leth-bridge sites was influenced by the Horse River Wildfire plumes.

## 3. Results and discussion

**Fig. 2:**
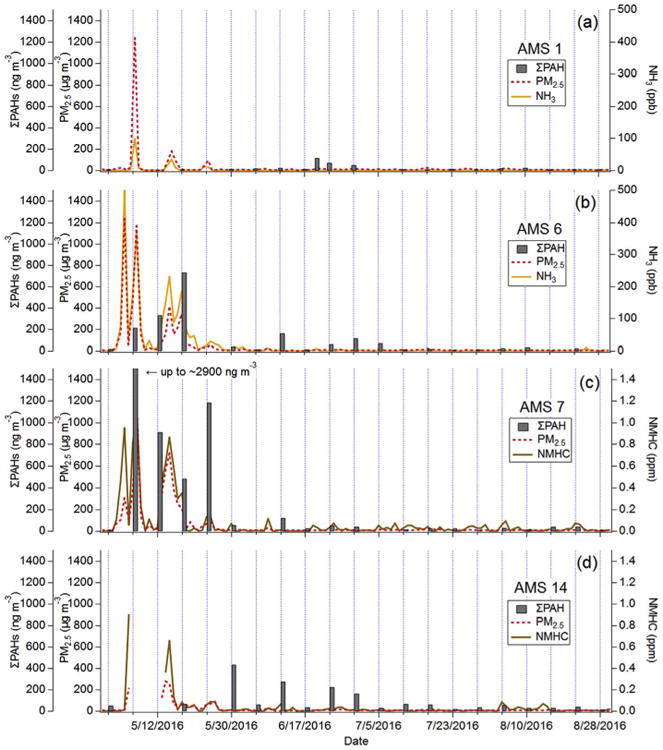
Time series of 24-h averaged ΣPAH (grey bars, left axis), PM_2.5_ mass (red dashed line, left axis), and NH_3_ (orange line, right axis) or NMHC (brown line, right axis). Measurements were taken at (a) AMS 1, (b) AMS 6, (c) AMS 7 and (d) AMS 14. (For interpretation of the references to colour in this figure legend, the reader is referred to the Web version of this article.)

“Fire-influenced” samples were defined as May samples taken on a day when the hourly average PM_2.5_ exceeded 25 μg m^−3^ for at least one hour during the 24-h PAH or VOC sampling window. The same PM_2.5_ threshold was used by Bytnerowicz et al. (2016) for distinguishing “fire periods” and “non-fire periods” during the 2011 Richardson fire in the AOSR. It is important to note that this distinction cannot account for the degree to which a sample was influenced, although a qualitative assessment can be made by examining ancillary measurements in Fig. 2. Furthermore, some days with all hourly averages below 25μg m^−3^ may still have some minor-to-moderate fire-influence. Table 1 summarizes the fire-influenced VOC and PAH samples taken during May 2016 at the seven AMS sites.

### 3.1. PAHs

Fig. 2 shows a time series of the sum of 23 PAHs (ΣPAH) at each site, as well as daily-averaged ancillary measurements (PM_2.5_, NH_3_, NMHC) to visually indicate periods inundated by wildfire smoke. Wildfire-influenced samples show a clear increase in ΣPAH by up to a factor ∼60 relative to non-fire periods (June to August). The average ΣPAH for fire-influenced samples (n = 8) was 852 ng m^−3^ (interquartile range = 300–980 ng m^−3^), compared to only 50 ng m^–3^ (interquartile range = 13–54 ng m^−3^) for non-fire influenced samples (n = 64). ΣPAH enhancement in fire-influenced samples ranged from 4 to 58 times, similar to the 3–65 factor increase reported by Aditama (2000) for a smouldering peat wildfire plume in Indonesia.

The maximum daily average ΣPAH was 2883 ngm^−3^ at AMS 7 on May 6th, shortly after the fire had passed through Fort McMurray on May 4th/5th when structure fires were prevalent throughout the city. The average fire influenced ^Σ^PAH concentration (852 ng m^−3^) far exceeded the 2012–2013 ΣPAH averages (∼ 10–30 ng m^−3^) previously reported at these sites (Hsu et al., 2015). However, Hsu et al. (2015) did not include naphthalene (NAP) in their total because of low collection efficiency of NAP in their study. The average fire influenced ΣPAH excluding NAP is 399 ng m^−3^ (interquartile range of 110–540 ng m^−3^), still well above observations by Hsu et al. (2015). The PAH sampling methodology for the WBEA network was altered in 2015 to improve collection efficiency for NAP, but is still likely below 100%.

In non-fire influenced samples, the average ΣPAH (without NAP) at AMS 1, 6 and 7 was 16ng m^−3^ (interquartile range of 6–18ng m^–3^), comparable to the ΣPAH concentrations reported by Hsu et al. (2015). The higher ΣPAH concentrations (upwards of 140 ng m^−3^ without NAP) at AMS 14 after May 30 are likely the result of local, temperature-dependent emissions of “volatile PAHs” near AMS 14, (Hsu et al., 2015). The enhancement of PAHs in fire-influenced samples was relatively consistent for volatile, semi-volatile, and particulate-bound PAHs (classifications defined by Hsu et al., 2015). A recent study by Schuster et al. (2015) measured bimonthly averages of 17 parent PAHs as well as alkylated PAHs at 17 sites throughout the AOSR for two years. Two bimonthly sampling periods in 2011 were influenced by forest fires with an increase in median ΣPAH by ∼2–5 times (upwards of ∼100ng m^–3^), which was more prevalent at sites closer to the flame front. The Schuster et al. (2015) and this study provide clear evidence of the considerable influence that boreal wildfires impart on PAH concentrations in the AOSR.

**Fig. 3:**
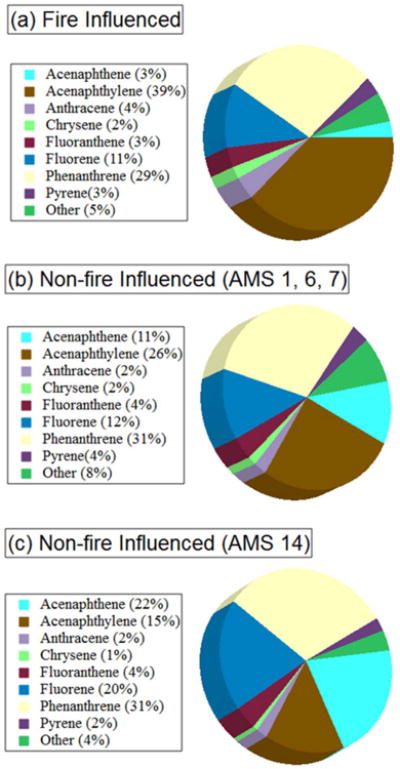
Average relative abundance of PAHs by mass, excluding NAP, for (a) fire-influenced samples at all sites, (b) non-fire influenced samples at AMS 1, AMS 6 and AMS7, and (c) non-fire influenced samples at AMS 14.

Fig. 3 shows the average relative abundance of the seven most prominent PAH species, excluding NAP which was between 30 and 80% of ΣPAH mass. These species were among the most abundant PAHs released from burn experiments of pine and fir biomass (Jenkins et al., 1996). The remaining 17 PAHs were each < 3% of the total ΣPAH mass in all samples. Overall the relative PAH abundances in fire influenced (Fig. 3a) and non-fire influenced (Fig. 3b and c) samples are largely similar. This implies that the emission profiles of major PAHs emitted from the wildfire were similar to other sources in the region. However, one key difference in Fig. 3 is the minor contribution of acenaphthene (ACE, ∼ 3%) in fire influenced samples relative to non-fire influenced samples (∼ 11% at AMS 1, AMS 6 and AMS 7; ∼ 22% at AMS 14). This suggests that the wildfire emitted much less ACE than acenaphthylene (ACY), fluorene (FLE), and phenanthrene (PHE), all of which had a much higher relative abundance in the fire influenced samples. Although there was an average 3-fold increase in ACE in fire influenced samples, increases for ACY (25-fold), FLE (16-fold) and PHE (15-fold) were much larger. This observation differs from Jenkins et al. (1996) who reported a higher emission factor for ACE than both ACY and FLE during controlled burn experiments of pine and fir under a variety of flaming conditions. This difference could be due to many confounding factors that affect PAH emission factors (e.g., flame intensity, oxygen availability, fuel type). AMS 14 was isolated from the other sites since it was likely influenced by different source(s) (Hsu et al., 2015). On average, there is a higher relative contribution of FLE and ACE at AMS 14 compared to the other three sites, consistent with the evidence from Hsu et al. (2015) showing local source(s) of high-volatility PAHs.

PAH diagnostic ratios have been used to aid in source identification in a wide variety of media (Akre et al., 2004; Tobiszewski and Namieśnik, 2012). Table 2 lists diagnostic PAH ratios used in previous studies to distinguish between various source types, although it should be noted that these studies include analyses of multiple media samples (e.g., air, sediments, soil). Despite their utility across different media, these diagnostic ratios are subject to large uncertainties due to the variability in source material, combustion conditions, and chemical processing; and should be considered qualitative as opposed to stringent quantitative thresholds (Galarneau, 2008; Zhang et al., 2005).

**Fig. 4:**
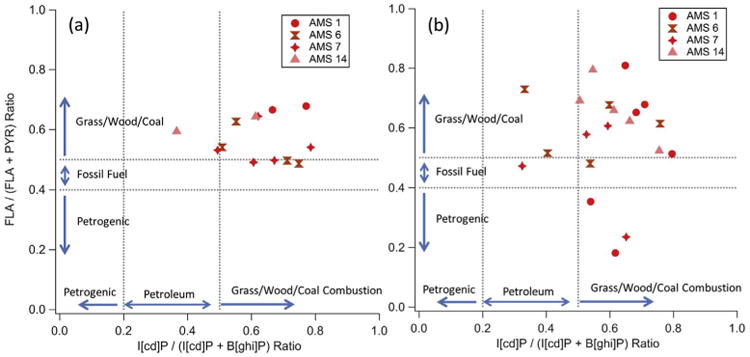
FLA/(FLA + PYR) versus I[cd]P/(I[cd] P + B[ghi]P) ratios for (a) May samples and (b) June 2016 samples. AMS sites are distinguished by marker shape and dashed lines denote the ratios used to differentiate various sources.

Fig. 4 shows each PAH sample taken in (a) May and (b) June at the four AMS sites plotted as a function of two PAH ratios from Table 2, in addition to the approximate threshold values commonly used to differentiate source types. All ratios in May (Fig. 4a), except one, are ∼ 0.5 or above which indicates the dominance of grass/wood/coal combustion. Furthermore, the median (mean) PHE/ANT ratio in fire-influenced samples was 7.6 (8.1) which implies a strong pyrolytic (incomplete combustion) signature. Fig. 4b reveals a wider spread of ratios in June (and frequently lower than ∼0.5) relative to May (Fig. 4a), suggesting more impact from non-coal fossil fuel combustion and petrogenic PAH sources. Furthermore, the median (mean) PHE/ANT ratio for June–August samples was 14.5 (14.4). However, there is evidence for contributions from lingering wildfire hotspots after May since the majority of samples in Fig. 4b still have ratios greater than 0.5.

For comparison, Evans et al. (2016) reported average summertime FLA/(FLA + PYR) and I[cd]P/(I[cd]P + B[ghi]P) ratios of 0.40 and 0.35, respectively, in air samples collected near upgrading facilities in the region. Similarly, Wnorowski (2017) observed an average phenanthrene to anthracene (PHE/ANT) ratio of 19 in air samples collected at AMS 13 in August and September 2013. Schuster et al. (2015) reported increasing FLA/(FLA + PYR) and [cd]P/(I[cd]P + B[ghi]P) ratios at sites further away from AOSR mining facilities. These previous studies show a dominant summertime influence of petrogenic and petroleum PAH sources throughout the AOSR. The shift of all three diagnostic ratios from petrogenic values (previous studies) towards pyrolytic/biomass burning values (this study) further reiterates the prevailing impact of the Horse River Wildfire on ΣPAH in May 2016. Hence, these PAH ratios show promise for being able to: (i) distinguish biomass burning from petrogenic influence, and (ii) constrain receptor models such as positive matrix factorization (Norris et al., 2014).

### 3.2. VOCs

**Fig. 5:**
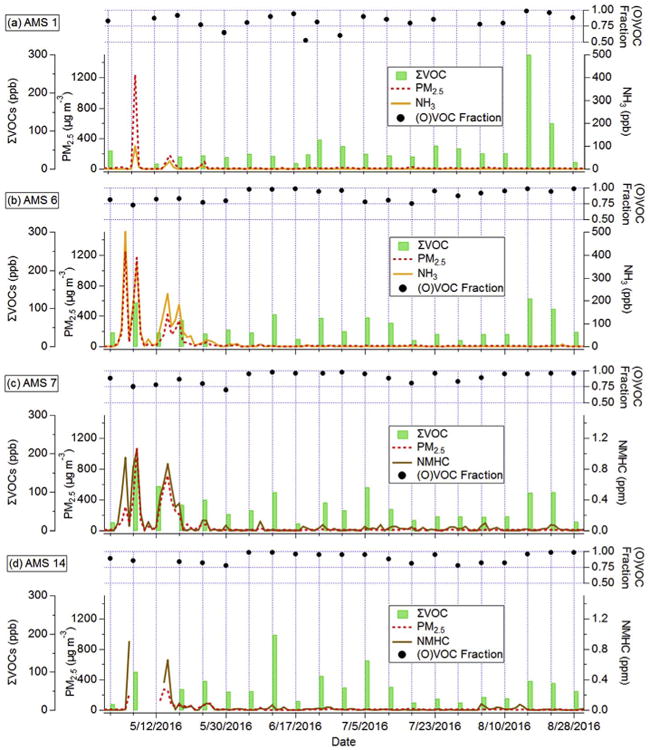
Time series of daily-averaged ΣVOC (green bars, left axis), PM_2.5_ (red dashed-line, left axis), and NH_3_ (orange line, right axis) or NMHC (brown line, right axis) for (a) AMS 1, (b) AMS 6, (c) AMS 7, and (d) AMS 14. The OVOC fraction (black circles) for VOC samples are shown at the top of each panel. (For interpretation of the references to colour in this figure legend, the reader is referred to the Web version of this article.)

Fig. 5 shows the sum of the 65 VOC mixing ratios (ΣVOC) at four sites, in addition to ancillary continuous measurements and OVOC (oxygenated VOC) fraction. Similar figures for the other three sites are in Supplemental Fig. S1. AMS 9 was omitted from the analysis below since it did not have a continuous PM_2_._5_ instrument. In contrast to the dramatic increase observed for ΣPAH in fire-influenced samples, there is only a minor-to-moderate relative increase in ΣVOC (Fig. 5). Fire-influenced samples (n = 16) had an average ΣVOC of 63 ppbv compared to 46 ppbv for the non-fire influenced samples (n = 90, excluding August 16th and 22nd, see Section 3.2.2). Interquartile ranges for the fire and non-fire influenced samples were 35–79 and 32–53 ppbv, respectively, showing considerable overlap and less variability compared to ΣPAH.

The average ΣVOC for fire-influenced samples at AMS 7 was 112 ppbv – about twice as high as any other site, which could be caused by its proximity to the fire and/or its location in a river valley. The high ΣVOC (average of 46 ppbv) in June to August is indicative of the multitude of biogenic and anthropogenic VOC sources in the AOSR (Li et al., 2017). The modest ΣVOC increase in fire-influenced samples is comparable to the modest increase observed by Ward et al. (2005) in Montana wildfire plumes. In both cases, a high non-fire VOC baseline may be obscuring the relative increase during fire-influenced periods.

**Fig. 6:**
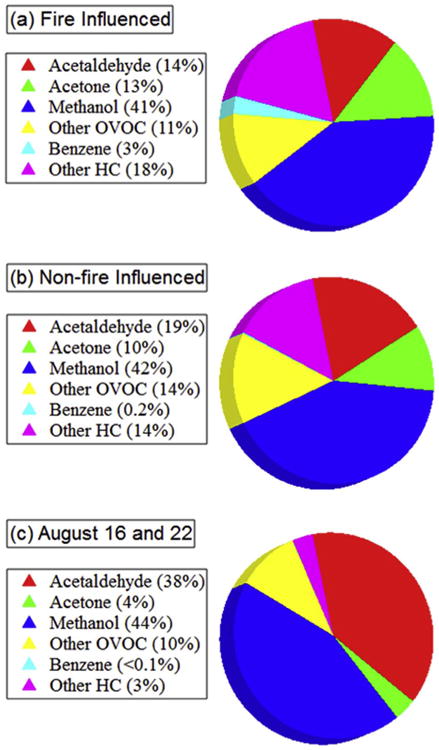
Average relative abundance of VOCs in percent v/v for (a) fire-influenced samples, (b) non-fire influenced samples excluding Aug 16 and 22, (c) the August 16 and 22 samples.

Fig. 6 shows the average relative abundance of major VOC species on fire influenced days (Fig. 6a), non-fire influenced days (Fig. 6b) and on August 16th and 22nd (Fig. 6c), in order to ascertain whether the VOC “fingerprint” differed between fire and non-fire influenced samples. The fire-influenced samples typically had lower OVOC fractions relative to the non-fire samples (average of 0.79 versus 0.86). Major VOC constituents in fire-influenced samples were methanol (37–44%, v/v), acetaldehyde (14–20%), acetone (8–15%), benzene (< 1–6%), 1-butene (< 1–4%), and formaldehyde (< 1–7%). These species are some of the most abundant VOCs emitted from extratropical forest fires and are broadly consistent with previously published emission factors (Andreae and Merlet, 2001; Urbanski, 2014). One exception is the minor contribution from formaldehyde, although this could be due to its short atmospheric lifetime. Other VOCs each contributed < 3% to measured ΣVOC.

The similarities between Fig. 6a and b and low contribution from HCs suggest significant atmospheric oxidation between emission and sampling. However, Yokelson et al. (1997) observed significant emissions of OVOCs including methanol, formaldehyde, acetic acid and formic acid during the heating (pre-combustion) and smouldering phases of controlled pine burning experiments. The smouldering phase involves less rigorous vertical mixing such that ground-level sampling is typically more sensitive towards smouldering conditions relative to flaming (Andreae et al., 1996). Acrolein, ethane, ethene, propyne, furan, formic acid, and acetic acid were not quantified but have emission factors from pine/spruce similar to, or greater than, methanol, acetaldehyde and acetone (Andreae and Merlet, 2001; Hatch et al., 2017). Further work is required to discern whether atmospheric processing and/or similarities in emission profiles between fire and non-fire sources resulted in comparable compositions dominated by OVOCs.

Fig. 5 and Figure S1 reveal high ΣVOC at all sites for the August 16th and August 22nd samples (average ΣVOC =123 ppbv). These mixing ratios were about twice as high as the fire-influenced samples, and between 2 and 5 times larger than other non-fire influenced samples. Furthermore, these two sampling days had: (i) no wildfire influence, (ii) very high OVOC fraction (average = 0.97), and (iii) all AMS sites affected. On average, methanol and acetaldehyde represented 82% of the measured ΣVOC (Fig. 6c). It is worthwhile to examine August 16th/22nd in detail since acetaldehyde exceeded the provincial air quality objective on these days (see Section 3.5).

In the summertime, acetaldehyde is produced primarily from photochemical reactions of ≥C3 alkenes, ≥C2 alkanes, ethanol, propanal and the decomposition of peroxyacetyl nitrate (PAN) (Luecken et al., 2012; Millet et al., 2010). Major sources of methanol include emission from vegetation, methane oxidation, and biomass decay, although the global methanol budget is poorly constrained (Heikes et al., 2002). HYSPLIT back-trajectories (Fig. S2) indicate that throughout August 16th air arrived from due west and passed over the western coast of British Columbia three days prior while remaining near the surface. In contrast, on August 22nd air was much more locally influenced and had spent the previous three days meandering near the surface within several hundred kilometres of Fort McMurray.

Due to disparate back-trajectories and plethora of acetaldehyde, methanol and precursor sources, it is unclear what the exact origin(s) of the August 16th and 22nd ΣVOC peaks are. However, the lack of wildfires in late August rules out wildfires as a potential source. It is also unlikely that biogenic emissions were the primary cause since isoprene, α-pinene, and β-pinene (the main biogenic precursors for acetaldehyde) were less than 3% of ΣVOC. To our knowledge, the only reported acetaldehyde measurements of comparably high concentrations are in areas with large anthropogenic VOC emissions, such as Mexico City (up to 99 ppb; Báez et al., 2000) or vast industrial complexes near Edmonton, Canada (up to 74 ppb; Simpson et al., 2013) and Gumi, South Korea (up to 77 ppb; Seo and Baek, 2011). It is important to note that these previous studies employed shorter integration times (3 h or less). In addition to direct emissions, facilities in the AOSR are known to be significant sources of acetaldehyde and methanol precursors (Li et al., 2017). Therefore, local photochemically aged anthropogenic VOCs were likely a contributing factor to the episodes of high acetaldehyde and methanol.

### 3.3. Enhancement ratios

Normalizing air pollutant concentrations to a relatively inert tracer gas, such as CO (atmospheric lifetime of ∼2 months), is a common method for assessing pollutant emissions from wildfires (e.g., Akagi et al., 2011; Hecobian et al., 2011; Landis et al., 2018; Simpson et al., 2011; Singh et al., 2010). These normalized excess enhancement ratios (ER) are calculated by dividing the excess of pollutant X above background (ΔX = X_plume_ – X_background_) by the excess of inert tracer Y (ΔY = Y_plume_ – Y_background_). For this study, hourly CO observations at AMS 7 were averaged to daily values to match the PAH and VOC observations. The median CO mixing ratio on non-fire influenced sampling days (117 ppbv) was used as the background CO value. Background mixing ratios for specific PAHs and VOCs were calculated in the same fashion. The CO monitor was offline for six hours on May 12th so this fire influenced sample is excluded from ER calculations.

Table 3 lists the ER (in ppb ppm^−1^) for the 20 VOC species which were above the detection limit in all three of the remaining fire influenced samples (May 6th, 18th, and 24th). For comparison, the average ER_X/CO_ from several aircraft studies that sampled VOCs in fresh boreal wildfire plumes are also given in Table 3.

The utility of the ER_VOC/CO_ values from this study may be limited by the small number of samples, which are reflected in the variability of the three samples. However, there are a greater number of VOCs investigated here relative to previous studies and the longer sample integration time may be more representative of emissions during various burning conditions and plume aging times. In general, the ER_VOC/CO_ values in this study are within a factor of 3 for VOCs that have been reported during short duration aircraft studies in boreal wildfire plumes (Hecobian et al., 2011; Simpson et al., 2011; Singh et al., 2010). These generally comparable ER_VOC/CO_ results provide some confidence in the range of ER_VOC/CO_ for species that have not yet been reported in the literature from ambient measurements of fresh boreal wildfire plumes. Nonetheless, additional measurements of ER_VOC/CO_ in fresh plumes would improve our understanding of VOC emissions from boreal wildfires.

To our knowledge, there are no reported ER_PAH/CO_ values reported in the literature for fresh boreal wildfire plumes. The values calculated for the fire-influenced samples at AMS 7 are given in Supplementary Table S4. The deficiency of ER_PAH/CO_ values for boreal wildfires highlights the need for additional measurements to better understand the emissions of PAHs from wildfires and their potential health effects.

### 3.4. O_3_ production downwind

Numerous studies have observed production of O_3_ downwind of large forest fires as a result of significant NO_x_ and VOC emissions (Brey and Fischer, 2016; Jaffe and Wigder, 2012; Morris et al., 2006; and referencies therein). Only a few studies have observed no effect of O3 in aged wildfire plumes (Alvarado et al., 2010; Tanimoto et al., 2008; Verma et al., 2009). These latter studies are almost exclusively in boreal regions and may be caused by a significant layer of optically-thick aerosols that hinders photochemistry (Verma et al., 2009) and/or the sequestration of NO_x_ as peroxyacetyl nitrate (PAN), which is more favourable at cooler temperatures (Alvarado et al., 2010).

**Fig. 7:**
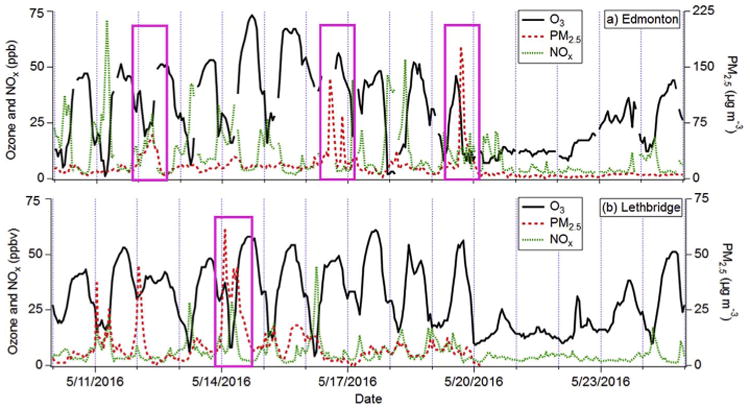
Continuous measurements of O_3_, PM_2.5_ mass, and NO_x_ at (a) Edmonton-Woodcroft and (b) Lethbridge during May 2016. Periods during which measurements were impacted by the Horse River Wildfire are highlighted by pink boxes. (For interpretation of the references to colour in this figure legend, the reader is referred to the Web version of this article.)

Fig. 7 examines four separate occasions (pink boxes) on which the Horse River Wildfire plume intercepted urban centres in Alberta after ∼ 0.5–2.5 days of travel. The influence from the wildfire was identified using ancillary continuous measurements from the Edmonton-Wood-croft (Fig. 7a) and Lethbridge (Fig. 7b) sites, as well as HYSPLIT back-trajectories and the FIRMS web mapper tool. HYSPLIT and FIRMS show that the two PM_2.5_ spikes on 11 May 2016 in Lethbridge were likely from a different wildfire in British Columbia.

Despite the presence of NO_x_ (Fig. 7) during the fire-influence periods, and an assumed increase in ΣVOC, there is no evidence for a substantial increase in O_3_ or O_x_ (≡; O_3_ + NO_2_). Although the small number of events prevented a quantitative analysis, O_3_ (O_x_) mixing ratios never went above 56 (60) ppbv as the wildfire plumes were advected through Edmonton and Lethbridge. There were no discernible changes in the typical diurnal O_3_ profile, nor was there a spike in O_x_ suggesting NO titration was minimal.

Meteorology was investigated using hourly data taken by Environment and Climate Change Canada at the Edmonton International (CYEG) and Lethbridge (CYQL) airports (ECCC, 2016). Table 4 summarizes the average air temperature, wind speed, and “weather” during the events. Rapid formation of O_3_ typically occurs under hot, stagnant, sunny conditions (Seinfeld and Pandis, 2016). All of the events were characterized by at least one of: cool average temperatures (< 7 °C), clouds/rain, and moderate-to-high wind speeds (≥8m s^−1^). These less-than-ideal conditions for O3 formation likely contributed to the absence of O_3_ enhancement. Cheng et al. (1998) presented a similar analysis for wildfire plumes from Northern Alberta passing through Edmonton from June 1–5, 1995. Although the authors measured increased NO_x_ and VOCs, they only observed enhanced O3 on June 4th and 5th. This was attributed to high winds from June 1–3 followed by hot, sunny and stagnant conditions on June 4–5.

Given that previous studies did not observe high O_3_ concentrations downwind of boreal fires (Alvarado et al., 2010; Tanimoto et al., 2008; Verma et al., 2009), and that less-than-ideal meteorological conditions for O_3_ formation persisted during plume intercepts, it is unsurprising that an O_3_ enhancement related to the Horse River Wildfire was not observed in Edmonton or Lethbridge. The influence of the 2016 Horse River Wildfire on transboundary O_3_ (∼1000s km away) was outside the scope of our study, but warrants further investigation.

### 3.5. Consequences for local air quality

Most epidemiological studies on wildfire smoke inhalation have focused their investigation on the impacts of particulate matter (PM) (Dennekamp and Abramson, 2011; Henderson and Johnston, 2012; Reisen and Brown, 2006; Youssouf et al., 2014; and references therein) while PAHs, VOCs and aldehydes have received far less attention, in part because of poorly defined and oftentimes lacking metrics. This section will focus on comparing ambient levels of PAHs and VOCs to air quality objectives and guidelines. An in-depth analysis of the fire's impacts on air quality from other pollutants (e.g., PM_2.5_, NO_x_, SO_2_) is the subject of a related manuscript (Landis et al., 2018).

Table 5 lists the 11 VOCs for which acute Alberta Ambient Air Quality Objectives (AAAQOs) exist (AEP, 2016c). A direct comparison to the hourly AAAQOs was complicated by the daily-averaged VOC measurements. Although not ideal, this approach is conservative since a daily average exceeding the hourly AAAQO likely had multiple hours which surpassed the metric. To provide more context, observations were also compared to the daily-averaged Minimal Risk Levels (MRLs) developed by the United States Agency for Toxic Substances & Disease Registry (ATSDR, 2016). Exposure above the AAAQOs and MRLs values does not mean negative health effects occurred. These values are set at levels which might cause adverse health effects in sensitive individuals and are simply intended to identify situations that require further examination.

Only acetaldehyde and benzene either approached or exceeded the AAAQO or MRL values during the May–August study period. The highest benzene values occurred during wildfire influenced periods and were always below 0.5 ppbv in non-fire influenced samples. Reinhardt and Ottmar (2004) measured average benzene levels of 16 ppb adjacent to wildfires and prescribed burns in the western United States. Both results suggest the need to better understand firefighter exposure to benzene and investigate potential health risks.

On the other hand, although acetaldehyde was enhanced during the wildfire (up to 27 ppbv at AMS 7 on May 6th), the highest mixing ratios were on August 16th and 22nd. For these two sampling days, all sites had daily average acetaldehyde above 24 ppbv with four of the fourteen samples exceeding the hourly AAAQO (50 ppbv). These acetaldehyde concentrations in the region were unprecedented. Historical VOC data from AMS 1 from 2009 to 2015 showed that the highest measured daily average acetaldehyde mixing ratio during this time was 23 ppbv, with the 98th percentile being 13 ppbv. Measurements of acetaldehyde in the AOSR should be conducted using a more common technique, such as DNPH-cartridge/HPLC (US EPA method TO-11A) or proton-transfer reaction mass spectrometry (PTR-MS). The evacuated canister GC/MS method in this study has a high detection limit (3 ppbv) and large uncertainty (± 24%). Nonetheless, offline canister-based methods have been used to measure acetaldehyde in numerous studies (e.g., Doskey, 2005; Rumsey et al., 2012; Simpson et al., 2013) and have shown good agreement with co-located samples collected using DNPH cartridges (Sistla and Aleksic, 2007).

This section is by no means a comprehensive analysis of the air quality implications from VOCs and PAHs during, or after, the Horse River Wildfire. Analysis was limited by several factors, including: (i) species not measured by the network, (ii) non-existent AAAQOs for most VOCs/PAHs, (iii) inconsistent time resolution for measurements and AAAQOs, and (iv) neglect of any potential synergistic effects of pollutants. Nonetheless, this section still identified the potential for acute health effects from benzene (during the wildfire) and acetaldehyde (after the wildfire) that merit further attention in subsequent studies on boreal wildfires, especially those in the AOSR.

## 4. Conclusions

This study examined routine measurements from an extensive air quality monitoring network in the AOSR both during and after the 2016 Horse River Wildfire. In May, sites were in close proximity (< 10 km) to the flame front which provided a unique situation to assess the impact of a large boreal fire on a considerable suite of air pollutants. Integrated PAH samples collected every sixth day revealed a 4–58 factor increase in ΣPAHs for fire-influenced samples. The major PAHs in these samples were NAP, ACY, PHE, FLE, and ACE. The relative proportion of the eight most abundant PAHs was similar between fire and non-fire influenced samples, with the exception of ACE which had a lower relative contribution to fire influenced samples. Diagnostic PAH ratios confirmed the significant impact of biomass burning at all four sites during May. After re-entry, these ratios provided evidence of PAHs from fossil fuel combustion and petrogenic origins, in addition to biomass burning from lingering wildfire hotspots.

In contrast, integrated VOC samples only had a minor increase for ΣVOC in fire-influenced samples (up to a factor of 3 greater than non-fire samples). The modest relative increase could be due, in part, to the plethora of VOC sources in the AOSR (Li et al., 2017; Simpson et al., 2010) that resulted in high ΣVOC concentrations during non-fire periods. Akin to the PAH samples, the relative contribution of the major VOCs (methanol, acetaldehyde and acetone) were comparable between fire and non-fire influenced samples. This phenomenon might be caused by significant atmospheric oxidation to form these short-chain OVOCs.

A ΣVOC increase larger than the wildfire-influenced samples occurred at all 7 sites on August 16th and 22nd. The cause(s) for these spikes are unknown, but were unique from the wildfire period due to (i) the lack of wildfires in the area, (ii) higher OVOC fraction, and (iii) presence at all monitoring sites. Due to the high OVOC fraction and abundance of acetaldehyde, aged anthropogenic emissions were likely a contributing factor.

Normalized excess enhancement ratios (ER) were calculated for 20 VOCs and 23 PAHs for three of the fire influenced samples at AMS 7. Results for VOCs were generally consistent with previously reported ER values in fresh North American boreal wildfire plumes. To our knowledge, this is the first study to report ambient ER values for PAHs in these types of plumes.

On four occasions in May 2016, the Horse River Wildfire plume passed through Edmonton or Lethbridge. Despite the presence of NO_x_ and a presumed increase in ΣVOC during these events, no appreciable O_3_ enhancement was observed. This finding is likely a consequence of meteorology unconducive for O_3_ formation on those days.

Benzene and acetaldehyde had daily averages that approached or exceeded the hourly AAAQO metric. Benzene was only significantly elevated in fire-influenced samples and may have posed an acute health risk for first responders. On the other hand, the highest mixing ratios of acetaldehyde were measured on August 16th and 22nd and exceeded the AAAQO in four samples. The sources, occurrence, and possible health impacts of acetaldehyde in the AOSR warrant further investigation, including measurements of higher time resolution to match the AAAQO averaging period of 1 h.

Higher frequency PAH and VOC sampling during wildfire events would help to better characterize peak concentrations and aid in assessing potential health risks to first responders. Samples should also be screened for as many fire-relevant VOCs, particularly acrolein which has a daily AAAQO of 0.17 ppb. PAH oxidation products (e.g., oxo- and nitro-polycyclic aromatic compounds) and retene (a common biomass burning marker) should also be measured. Monitoring CO and CO_2_ would allow for calculation of the modified combustion efficiency (MCE) and would help determine which phases of the wildfire were most detrimental for degrading air quality.

## Supplementary Material

Supp 1

## Figures and Tables

**Table 1:** Fire-influenced integrated samples.

Date	AMS 1	AMS 6	AMS 7	AMS 9*	AMS 13	AMS 14	AMS 15
May 6	Yes	Yes	Yes	N/A	Yes	–	Yes
May 12	No	Yes	Yes	N/A	No	–	No
May 18	No	Yes	Yes	N/A	No	Yes	Yes
May 24	Yes	Yes	Yes	N/A	Yes	Yes	Yes
May 30	No	No	No	N/A	No	No	No

* N/A since AMS 9 samples VOCs but does not have a continuous PM_2.5_ monitor.

‘-‘ indicates PM_2.5_ instrument was offline.

**Table 2:** Diagnostic PAH ratios and the typical range for source types.

PAH Ratio	Threshold	Source Type	References
FLA/(FLA + PYR)	< 0.4	Petrogenic	De La Torre-Roche et al. (2009); Tobiszewski and Namieśnik (2012)
	0.4–0.5	Fossil Fuel	
	> 0.5	Combustion Grass, Wood, Coal Combustion	
I[cd]P/(I[cd]P + B [ghi]P)	< 0.2	Petrogenic	Tobiszewski and Namieśnik (2012); Yunker et al. (2002)
	0.2–0.5	Petroleum	
	> 0.5	Combustion Grass, Wood, Coal Combustion	
PHE/ANT	<10	Pyrolytic	Baumard et al. (1998); Yang (2000); Wnorowski (2017)
	>15	Petrogenic	

FLA =fluoranthene, PYR = pyrene, I[cd]P=indeno[1,2,3-c,d]pyrene, B[ghi]P =benzo [g,h,i]perylene, PHE = phenanthrene, ANT = anthracene.

**Table 3:** Comparison of VOC Enhancement Ratios (in ppb ppm^−1^ CO) for fire influenced samples at AMS 7 to literature values for fresh North American boreal forest wildfires.

VOC	May 6	May 18	May 24	Average	Simpson et al. (2011)	Hecobian et al. (2011)	Singh et al. (2010)
1,3-Butadiene	0.36	0.15	0.29	0.27	–	–	–
1-Butene	1.50	0.67	2.2	1.5	–	–	–
1-Pentene	0.18	0.069	0.27	0.17	–	–	–
Acetaldehyde	4.1	1.0	7.9	4.3	–	–	4.9 ± 3.3
Acetone	2.6	2.6	6.3	3.8	1.6 ± 0.4	–	4.7 ± 3.3
Benzene	2.0	0.91	3.7	2.2	1.7 ± 0.3	1.3 ± 0.5	1.6 ± 0.3
cis-2-Butene	0.18	0.074	0.27	0.17	–	–	–
Ethylbenzene	0.082	0.010	0.19	0.094	0.058 ± 0.02	–	–
Isobutane	0.53	0.28	0.97	0.59	–	–	–
Isopentane	0.18	0.089	0.16	0.14	–	–	–
m,p-Xylene	0.18	0.015	0.29	0.16	–	–	–
Methanol	13	2.0	9.5	8.2	9.6 ± 1.9	–	15.6 ± 9.2
Methylethylketone	0.66	0.30	0.95	0.64	0.38 ± 0.1	–	–
n-Butane	0.76	0.32	1.5	0.86	0.32 ± 0.05	–	–
n-Octane	0.22	0.015	0.17	0.14	–	–	–
n-Pentane	0.23	0.049	0.16	0.15	0.14 ± 0.02	–	–
o-Xylene	0.071	0.0050	0.14	0.072	–	–	–
Toluene	1.0	0.33	2.1	1.1	0.67 ± 0.16	0.6 ± 0.3	0.7 ± 0.2
trans-2-Butene	0.23	0.069	0.32	0.21	–	–	–
trans-2-Pentene	0.052	0.015	0.095	0.054	–	–	–

**Table 4:** Average meteorological conditions during Horse River Wildfire plume events in Edmonton and Lethbridge.

Location	Duration (Local Time)	Temp (°C)	Wind Speed (m s^−1^)	Weather
Edmonton	May 12–0:00 to 12:00	6.5	3.0	Cloudy
Edmonton	May 16–9:00 to 22:00	3.8	3.3	Cloudy
Edmonton	May 19–16:00 to 0:00	22.6	8.2	Clear
Lethbridge	May 14–1:00 to 12:00	5.5	8.0	Rain

**Table 5:** AAAQOs, MRLs and highest daily averages (May–Aug, inclusive) for 11 VOCs.

Substance	AAAQO (1-hr, ppbv)	MRL (24-hr, ppbv)	Highest Daily Average (ppbv, May–Aug 2016)
Acetaldehyde	50	N/A	196
Acetone	2400	26,000	15
Benzene	9.0	9.0	8.7
Ethylbenzene	460	5000	1.7
Formaldehyde	40	53	24
Isopropylalcohol	3190	N/A	2.2
Methanol	2000	N/A	162
n-hexane	5960	600	15
o-xylene	530	N/A	1.5
Styrene	52	N/A	0.3
Toluene	499	2000	4.6
