# Supplementary material for: Impacts of a large boreal wildfire on ground level atmospheric concentrations of PAHs, VOCs and ozone
Source: Atmos Environ (1994). Author manuscript; Available in PMC 2018 May 1. (PMC5906807; doi:10.1016/j.atmosenv.2018.01.013)
Supplement: Supp 1 [file NIHMS954541-supplement-Supp_1.docx]

Supplemental

Table S1. Method detection limits for PAHs measured at the AMS sites

| **PAH** | **Method Detection Limit (ng m^-3^)** |
| --- | --- |
| 3-Methylcholanthrene3 | 0.022 |
| 7,12-Dimethylbenz(a)anthracene3 | 0.013 |
| Acenaphthene | 0.0064 |
| Acenaphthylene | 0.011 |
| Acridine2 | 0.019 |
| Anthracene | 0.017 |
| Benz(a)anthracene | 0.014 |
| Benzo(a)pyrene | 0.016 |
| Benzo(b)fluoranthene | 0.020 |
| Benzo(c)phenanthrene | 0.015 |
| Benzo(ghi)perylene | 0.020 |
| Benzo(k)fluoranthene | 0.013 |
| Chrysene | 0.013 |
| Dibenz(a,h)anthracene | 0.020 |
| Dibenzo(a,h)pyrene | 0.020 |
| Dibenzo(a,i)pyrene | 0.025 |
| Dibenzo(a,l)pyrene | 0.024 |
| Fluoranthene | 0.0070 |
| Fluorene | 0.0071 |
| Indeno(123-cd)pyrene | 0.017 |
| Naphthalene | 0.0082 |
| Phenanthrene | 0.0071 |
| Pyrene | 0.0083 |

Table S2. Method detection limits for VOCs measured at the AMS sites

| VOC | Method Detection Limit (ppbv) |
| --- | --- |
| 1,2,4-Trimethylbenzene | 0.03 |
| 1,3,5-Trimethylbenzene | 0.02 |
| 1,3-Butadiene | 0.02 |
| 1-Butene | 0.02 |
| 1-Pentene | 0.01 |
| 2,2,4-Trimethylpentane | 0.01 |
| 2,2-Dimethylbutane | 0.01 |
| 2,3,4-Trimethylpentane | 0.01 |
| 2,3-Dimethylbutane | 0.02 |
| 2,3-Dimethylpentane | 0.02 |
| 2,4-Dimethylpentane | 0.01 |
| 2-Methyl-1-pentene | 0.3 |
| 2-Methyl-2-butene | 0.3 |
| 2-Methylheptane | 0.01 |
| 2-Methylhexane | 0.01 |
| 2-Methylpentane | 0.01 |
| 3-Methyl-1-butene | 0.3 |
| 3-Methylheptane | 0.02 |
| 3-Methylhexane | 0.02 |
| 3-Methylpentane | 0.01 |
| 4-Methyl-1-pentene | 0.3 |
| Acetaldehyde* | 3 |
| Acetone* | 0.4 |
| α-Pinene | 0.3 |
| Benzene | 0.01 |
| β-Pinene | 0.3 |
| cis-2-Butene | 0.02 |
| cis-2-Hexene | 0.3 |
| cis-2-Pentene | 0.02 |
| Cyclohexane | 0.02 |
| Cyclopentane | 0.01 |
| Cyclopentene | 0.3 |
| Ethanol* | 0.3 |
| Ethylbenzene | 0.01 |
| Formaldehyde* | 3 |
| Isobutane | 0.02 |
| Isobutylene | 0.3 |
| Isopentane | 0.03 |
| Isoprene | 0.01 |
| Isopropylalcohol* | 0.4 |
| Isopropylbenzene | 0.01 |
| m,p-Xylene | 0.03 |
| Methanol* | 3 |
| Methylcyclohexane | 0.01 |
| Methylcyclopentane | 0.02 |
| Methylethylketone* | 0.3 |
| Methylisobutylketone* | 0.4 |
| Methylvinylketone* | 0.3 |
| n-Butane | 0.03 |
| n-Decane | 0.06 |
| n-Dodecane | 0.4 |
| n-Heptane | 0.01 |
| n-Hexane | 0.01 |
| n-Nonane | 0.01 |
| n-Octane | 0.02 |
| n-Pentane | 0.1 |
| n-Propylbenzene | 0.05 |
| n-Undecane | 0.5 |
| Naphthalene | 0.5 |
| o-Xylene | 0.01 |
| Styrene | 0.04 |
| Toluene | 0.01 |
| trans-2-Butene | 0.01 |
| trans-2-Hexene | 0.3 |
| trans-2-Pentene | 0.02 |

*denotes OVOC

Table S3. Instrument methods and detection limits for continuous ambient data

| Analyte | Instrumental Method | Approximate Detection Limit* |
| --- | --- | --- |
| PM_2.5_ mass | TEI SHARP 5030 | 0.5 µg m^-3^ |
| NH_3_ | TEI 17C or TEI 17i | 1 ppbv |
| NMHC | TEI 55i | 50 ppbv |
| THC | TEI 51 CLT, TEI 51 iLT, or Rosemount 400A | 0.05 ppm |
| NO_x_ | TEI 42C, TEI 42i, or Teledyne API 200A | 1 ppbv |
| O_3_ | TEI 49C, TEI 49i, or Teledyne API 400A | 1 ppbv |
| CO | TEI 48C | 0.05 ppm |

*Varies depending on status of individual analyzer

Table S4. PAH Enhancement Ratios (ng m^-3^ ppm^-1^ CO) for fire influenced samples at AMS 7

| **PAH** | **May 6** | **May 18** | **May 24** | **Average** |
| --- | --- | --- | --- | --- |
| 3-Methylcholanthrene | 0.18 | 0.15 | 0.21 | 0.18 |
| 7,12-Dimethylbenz(a)anthracene | 2.4 | 0.47 | 2.9 | 1.9 |
| Acenaphthene | 19 | 1.4 | 36 | 19 |
| Acenaphthylene | 110 | 33 | 760 | 300 |
| Acridine | 1.9 | 0.31 | 0.91 | 1.0 |
| Anthracene | 13 | 2.8 | 9.5 | 8.5 |
| Benz(a)anthracene | 5.9 | 0.56 | 3.1 | 3.2 |
| Benzo(a)pyrene | 2.1 | 0.28 | 1.0 | 1.1 |
| Benzo(b)fluoranthene | 3.4 | 0.37 | 0.89 | 1.6 |
| Benzo(c)phenanthrene | 1.7 | 0.28 | 1.3 | 1.1 |
| Benzo(ghi)perylene | 1.1 | 0.14 | 0.16 | 0.45 |
| Benzo(k)fluoranthene | 2.2 | 0.28 | 0.93 | 1.1 |
| Chrysene | 12 | 1.4 | 7.3 | 6.7 |
| Dibenz(a,h)anthracene | 0.66 | 0.13 | 0.59 | 0.46 |
| Dibenzo(a,h)pyrene | 0.038 | 0.032 | 0.061 | 0.044 |
| Dibenzo(a,i)pyrene | 0.041 | 0.18 | 0.041 | 0.087 |
| Dibenzo(a,l)pyrene | 0.20 | 0.10 | 0.055 | 0.12 |
| Fluoranthene | 19 | 1.7 | 11 | 10 |
| Fluorene | 92 | 4.2 | 30 | 42 |
| Indeno(123-cd)pyrene | 1.0 | 0.22 | 0.41 | 0.55 |
| Naphthalene | 250 | 150 | 840 | 410 |
| Phenanthrene | 99 | 26 | 120 | 82 |
| Pyrene | 16 | 1.7 | 11 | 9.6 |


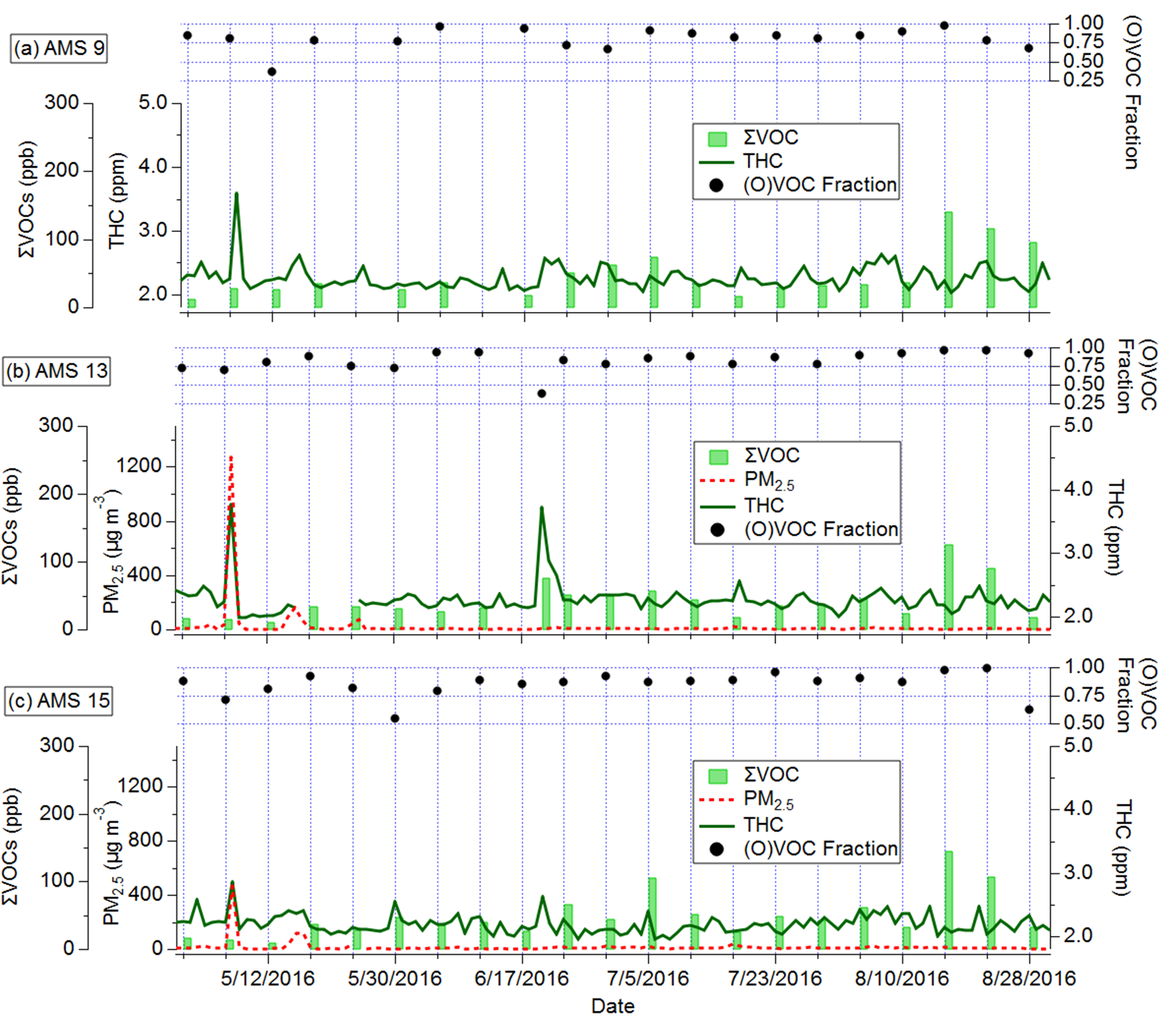


Figure S1. Time series of daily-averaged ΣVOC, PM­_2.5_, and THC at (a) AMS 9, (b) AMS 13, and (c) AMS 15. The calculated OVOC fraction (black circles) for VOC samples are shown at the top of each panel.


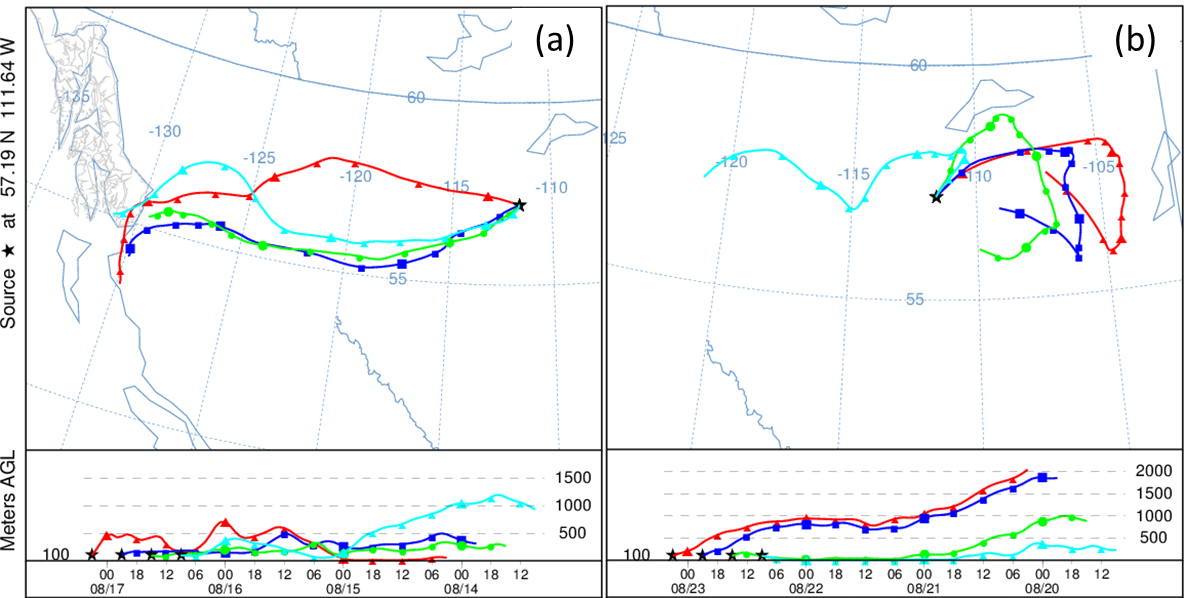


Figure S2. HYSPLIT back-trajectories arriving at AMS 1 on (a) 16 August 2016, and (b) 22 August 2016 at 100 m. Back-trajectories were calculated for local arrival times of 3:00 (light blue), 9:00 (green), 15:00 (dark blue), and 21:00 (red).
